# Towards a framework for business model innovation in health care delivery in developing countries

**DOI:** 10.1186/s12916-014-0233-z

**Published:** 2014-12-02

**Authors:** Ramon Castano

**Affiliations:** Organización para la Excelencia de la Salud, Bogota, Colombia

**Keywords:** Business model innovation, Quality care, Standardization, Separability, Patient-centeredness

## Abstract

**Background:**

Uncertainty and information asymmetries in health care are the basis for a supply-sided mindset in the health care industry and for a business model for hospitals and doctor’s practices; these two models have to be challenged with business model innovation. The three elements which ensure this are standardizability, separability, and patient-centeredness. As scientific evidence advances and outcomes are more predictable, standardization is more feasible. If a standardized process can also be separated from the hospital and doctor’s practice, it is more likely that innovative business models emerge. Regarding patient centeredness, it has to go beyond the oversimplifying approach to patient satisfaction with amenities and interpersonal skills of staff, to include the design of structure and processes starting from patients’ needs, expectations, and preferences. Six business models are proposed in this article, including those of hospitals and doctor’s practices.

**Discussion:**

Unravelling standardized and separable processes from the traditional hospital setting will increase hospital expenditure, however, the new business models would reduce expenses. The net effect on efficiency could be argued to be positive. Regarding equity in access to high-quality care, most of the innovations described along these business models have emerged in developing countries; it is therefore reasonable to be optimistic regarding their impact on access by the poor. These models provide a promising route to achieve sustainable universal access to high quality care by the poor.

**Summary:**

Business model innovation is a necessary step to guarantee sustainability of health care systems; standardizability, separability, and patient-centeredness are key elements underlying the six business model innovations proposed in this article.

## Background

Health care systems face a growing pressure to meet people’s needs with limited resources. The pressure grows, among other reasons, as a consequence of demographic and epidemiologic transitions, but mostly as a consequence of new medical technologies that generate small incremental benefits at high incremental costs [1]. This begs the question, why is it that health care technologies increase costs instead of the opposite, as is the case in other industries? Several authors have proposed that health systems should create more value for money [2] or that they should have a triple aim of better health, better care, and lower costs [3]. Thus, why do these undisputable goals prove so elusive and health expenditures continue to grow apparently irrespective of value creation? What are the root causes of this apparently uncontrollable problem of sustainability?

### The root causes of the problem of sustainability

In a seminal paper in health economics, Kenneth Arrow proposed that uncertainty in the diagnosis and treatment of disease makes it difficult for doctors and hospitals to achieve predictable outcomes [4]. In addition, large information asymmetries between doctors and patients make it difficult for the latter to drive quality improvements and innovations that yield more value for less money [4]. On the other hand, those societies that recognize a right to health care, isolate the individual’s willingness or ability to pay from the actual medical care received. This creates a moral hazard problem whereby the marginal benefit of care is far lower than its marginal cost. As a consequence of uncertainty and information asymmetry, non-market institutions emerge to control the potential agency problems that markets or government regulations cannot control by themselves. Professionalism, not-for-profit status of hospitals, prohibition of advertising and price competition, and lack of an obligation to guarantee good outcomes, are at the heart of medical care values, according to Arrow [4].

These values might seem obvious to people within the health care industry. However, contrasting this industry with others where uncertainty and information asymmetry are not that large, sheds some light about the fundamental problems of current health care systems. Although uncertainty and information asymmetries always exist, it is clear that in markets such as, e.g., personal computers or mobile phones, consumers have enough information and certainty to make choices based on their preferences and willingness to pay and net of risks. Value for money is the driver of competition, quality improvements and innovation, and producers are pushed every day to create new products that yield more value at lower prices. Just think of the price you paid for a laptop computer 10 years ago and its performance as compared to your current computer.

In health care, value is not so obvious to patients. Although every patient wants to have better quality of life and better functional capacity (broadly addressed through the increasingly used concept of patient-reported outcomes), it is less obvious how the patient would assess the short-term clinical outcomes that yield those benefits. Hence the doctor’s role as an agent for the patient, in order to fill the information gaps. As a consequence, doctors and health care providers have been the traditional designers of solutions to patients, which gives them the privilege to create the structures and processes that, provided good compliance to clinical practice, are expected to result in the best possible outcomes. However, patients themselves are isolated from such design, except for those quality attributes that are observable for them, such as amenities and staff interpersonal skills. This role of doctors and providers as designers of solutions gives them a supply-sided mindset. In other industries that are driven by consumers’ search for value for money, the design of value propositions starts from consumers’ needs, expectations, and preferences, i.e., producers have a consumer-driven mindset. Perhaps this is the most basic difference that, based on Arrow’s powerful insights [4], explains why the health care industry lags behind other industries in creating more value for each dollar spent.

However, doctors and providers do not deliberately ignore that creating value for patients is a paramount goal for their day to day work. The problem lies in how value is defined. Value in health care should be defined as better outcomes for each dollar spent, and outcomes should be defined around patients, not just around compliance with processes, or around a number of services provided or a simplistic view of patient satisfaction [2]. This begs the question, why is it not possible for doctors and health care providers to guarantee better outcomes to such an extent that value-based competition works the way it does in other industries? According to Christensen et al. [5], another fundamental reason, rooted in the same dysfunctionalities described by Arrow, is the business model of hospitals and doctors’ practices. The authors point to the fact that these two business models are adequate to deal with uncertainty, but when uncertainty is reduced and outcomes become more predictable, different business models must be developed to exploit the advantages of standardization and task shifting [5]. During the early twentieth century, when these two business models (as we know them today) first emerged, most health care was uncertain. The general hospital and the doctor’s practice were adequate to the nature of the task because they allowed for a wide array of resources and capabilities to deal with the multiple contingencies that are typical of uncertain processes. However, to the extent that scientific evidence improves and outcomes become more predictable, uncertainty is reduced for some health care services. Consequently, the better the evidence, the more predictable the outcomes, and the more likely it will be to streamline the structures and processes that are necessary for these types of services. Streamlining means standardization of processes, and once standardized, those processes can in many instances be delegated to other staff. It is worth noting that standardization by itself does not assure the widespread adoption of the standard, a problem reported by McGlynn et al. [6].

According to Christensen et al. [5], a major stumbling block in the road to more value for money is that general hospitals and doctor’s practices mix both standardized and non-standardized processes in the same business model. If standardized processes can be separated from non-standardized ones, the former can be organized in more efficient ways and outcomes will improve because of their high predictability. To the extent that these processes are kept together with non-standardized processes, the variability and uncertainty of the latter will affect the predictability of the standardized processes. It appears then, that the structure of health care delivery is at the root of the problems most health care systems face. It is not that doctors and health care providers are incompetent or deliberately poor performers. Rather, it is the business models underlying medical care which need to be changed. This claim has been summarized by Ashish Jha, who says that “…*we deliver 21*^*st*^*century medicine using 19*^*th*^*century practices*” [7]. Porter and Teisberg point that “…*the structure of health care delivery is the most fundamental issue*” [2]. Christensen et al. claim that “*The lack of business model innovation in the health-care industry—in many cases because regulators have not permitted it—is the reason health care is unaffordable*” [5]. Moreover, McGlynn et al.’s claims regarding unacceptably low rates of adoption of standards, can be interpreted as symptoms of the structural flaws of the prevailing business models [6].

Many commentators have argued that the problem lies in how doctors and health care providers are paid. Fee-for-service reimbursement creates incentives for demand inducement, and no incentives to achieve better outcomes through care coordination across the entire cycle of care of a given medical condition. The lack of incentives for coordination leads to severe fragmentation of care, which makes it impossible for any provider to be accountable for outcomes when these require coordination among several providers. The quick reaction to the bad consequences of fee-for-service has been to shift towards prospective payment modalities that transfer risk to providers, on the expectation that this will create the incentives to avoid demand inducement and to improve coordination of care.

However, when doctors and providers switch from retrospective to prospective payments but do not change their business models, the perverse incentives associated with fee-for-service (demand inducement, lack of coordination) are replaced with the perverse incentives associated with capitation (cost shifting, skimping on care, and cream skimming). In health systems where providers compete for patients or for contracts with payers, and in the absence of business model changes, both retrospective and prospective payments lead to value destruction at both the provider and the payer level. In health systems without competition and lack of business model changes, value destruction also ensues as a consequence of how providers are paid or how they receive their operating budgets. Therefore, payment mechanisms by themselves do not create enough incentives to increase value. A more fundamental change has to take place: a radical change in business model.

### Key elements for business model innovation

It could be argued that standardizability is an important element enabling innovations in business models followed by separability from hospital, and, finally, patient-centered innovation.

Standardizability depends on knowledge about how a process can be structured to achieve an outcome with minimal uncertainty. Therefore, the stronger the scientific evidence explaining how *A* causes *B*, the more likely the process will be standardized: the best way to make sure that the expected outcome (*B*) is achieved, is making sure the process (*A*) is strictly followed [8]. Standardization is a necessary condition for task-shifting. A complex task that is surrounded by uncertainty requires judgment based on expertise and experience. Therefore, it has to be performed by a highly skilled worker. In the opposite sense, a simple task with little or no uncertainty requires little judgment and can be written in a protocol, which can be delegated to a less skilled worker. The most extreme case of delegation is an automatic task that is delegated to a computer or a machine. A good example of an entire process that has been standardized, and therefore delegated, is the application of an immunization schedule for a baby. However necessary, standardization is not a sufficient condition for delegation. Not all that is standardizable can be delegated to less skilled workers. For example, placing a stent in a coronary artery or performing a total hip replacement requires a learning process that cannot be leapfrogged by learning a protocol.

Separability is the principle whereby a given process does not depend on a wide array of equipment, supplies, and workforce, and can be set up in a separate facility with dedicated resources that can be efficiently used, i.e., with little or no idle capacity. Interdependencies in hospital-based processes are typical of complex patients, which makes these processes less separable. For example, a given patient with metastatic lung cancer will require many diagnostic and therapeutic processes and surgical and non-surgical procedures, all requiring a wide variety of specialties, equipment, and consumables. Another patient with the same type of cancer might need a different mix of inputs, depending on the particular characteristics of that patient. Therefore, interdependencies make it very difficult to separate care for patients with metastatic lung cancer in a freestanding facility.

A process of care that is highly standardized and highly separable from a hospital or a doctor’s practice is more likely to be arranged in an innovative business model that widely differs from these two traditional business models. Examples of this are cataract surgery or hernia repair, as performed at Aravind Eye Care System [9] and Shouldice Hospital [10], respectively. Nevertheless, these two models have been on stage for several decades, which hardly makes them innovative. The interesting question is why they are not more pervasive in health care systems. One business model that is enabled by both standardizability and separability is that of specialized community health workers. In this model, lay people apply highly standardized protocols for following up patients in post-acute phase and patients with chronic conditions in their community setting. Community workers do not make clinical decisions; they are supervised by doctors or nurses, who make clinical decisions and use them as their “extensors” [11]. Nonetheless, non-standardizable and non-separable processes can also be reshaped with innovative business models as will be shown below. This means that business model innovation is also possible within the current hospital and doctor’s practice models.

The third key element for innovation is patient centeredness, or more broadly, person centeredness. As said above, the supply-side mindset of health care providers makes it difficult for them to understand patients’ needs, expectations, and preferences, and to see them as opportunities for innovation. The farthest that most providers go into this realm is to focus on patient satisfaction with facilities and interpersonal skills of staff. However, patient-centeredness goes far beyond this oversimplifying approach. A case in point is care for rheumatoid arthritis. Two therapeutic goals are key in caring for these patients: prevention of structural damage and control of symptoms; abrogation of inflammation achieves these two goals [12]. According to the author’s own unpublished research in Bogota, Colombia, from many doctors’ perspective, it is enough to see the patient every one to three months to keep track of levels of disease activity. However, when a patient has a flare, she wants to see the doctor immediately. However, the typical business model of an ambulatory care center is not adequate to deal with non-scheduled visits, and to deal with the processes and activities required for the short-term management of flares. Therefore, patients must attend an emergency room or visit another doctor, with the consequent fragmentation of the cycle of care and coordination problems. Managing short-term symptoms is much more important from the patient perspective than from the doctor’s. However, a business model that is designed from the doctor’s perspective to achieve long-term goals does not meet patient needs, expectations, and preferences in the short term.

This case illustrates the gap between a supply-sided mindset and what consumers need, expect, or prefer. It does not matter how well the staff treats the patient in terms of interpersonal manners or how sympathetic they are to patient complaints. It does not matter how convenient the facility is designed in terms of physical access, waiting rooms, and amenities. The real problem is that the whole business model has to be redesigned to be able to deal with how the patient experiences illness. The traditional supply-sided mindset of doctors and health care providers is, in fact, a tremendous opportunity for business model innovation, as illustrated with the rheumatoid arthritis case. Most other industries have become very sophisticated in anticipating consumer’s needs, expectations, and preferences in order to come up with new value propositions to win the race for consumer votes. This head-to-head race has led to repeated disruptions that have yielded much better products and services at lower prices. Health care lags far behind those industries, so the opportunities for innovation are immense, just by turning back to patients and understanding their needs, expectations, and preferences in a much deeper way than the oversimplifying traditional approach to patient satisfaction.

### Six business models instead of one

The process of care can be simplified into three steps: diagnosis, treatment, and self-care/self-management. Each of these steps has both standardized and non-standardized components. A simple way to understand how standardizable and non-standardizable processes allow for innovation in health care delivery models is to depict them as six separate building blocks that can be rearranged in different patterns. Table 1 illustrates this framework. These six blocks are mixed in the current business models of hospitals and doctors’ practices, which is partly the source of their dysfunctionality, as shown above.

**Table 1:** **The six building blocks for innovative health care delivery models**

	**Diagnosis**	**Treatment**	**Self-care/self-management**
Standardized	Block 1	Block 2	Block 3
Non-standardized	Block 4	Block 5	Block 6

Source: author’s creation.

The framework in Table 1 allows for six separate categories, each with its own business models, as follows:

- Medical conditions for which diagnosis and treatment are highly standardized (blocks 1 and 2). This category includes, for example, diarrhea, minor sunburns, athlete’s foot, etc. These conditions are currently the focus of retail clinics in the United States [13] and telephone assistance business models. In these two models, the process of care is standardized, can be delegated to non-physicians, and operates outside hospitals and doctors’ practices. The business model of community health workers cited above is another example of this category.
- Medical or surgical treatments that are highly standardized such as hernia repairs, cataract surgery, or kidney transplantation (block 2). In this category, some or all activities are highly standardized, and some of the most standardized ones are delegated to non-physicians [9]. Although cataract surgery and hernia repairs are highly separable from general hospitals, kidney transplantation is not separable, particularly the surgical procedure. The high level of standardization of these processes has deserved them the name “focused factories” [14]. When the process is not separable from the general hospital, the figure of “a hospital within a hospital” allows for keeping interdependencies while avoiding the interference of non-standardized processes [8].
- Diagnostic processes (block 4). In this category, the idea is to separate the diagnostic process from the treatment decision. This separation aims to avoid the framing bias that makes it more likely that a doctor interprets signs and symptoms through the lens of those medical conditions he or she knows how to treat, or is familiar with. Although it could be argued that a diagnostic algorithm is in fact a standardized process, it is clear that, for a given patient, it cannot be anticipated which of the diagnostic possibilities she has, until the process of hypothesis testing advances through the algorithm. To avoid framing bias, the diagnostic process should be performed in an interdisciplinary way and an explicit process has to be put in place to force clinicians to share their perspectives before making a decision about diagnosis.
- Self-care and self-management (blocks 3 and 6). This category includes many processes that are highly standardized, such as taking medications, applying non-pharmacological therapies, changing habits, and adopting healthy lifestyles. Other processes, such as responding to the particular psychological or social needs of a given patient, are not standardizable. Both processes can be dealt with more effectively through peer-support communities [15]. Christensen et al. propose the business model of facilitated networks: web-based or real groups of people that share common interests [5]. In this case, the common interest is a medical condition, and the network members support themselves to reinforce positive behaviors and discourage negative ones. The classic example of this category is Alcoholics Anonymous, but modern-time facilitated networks have become pervasive thanks to the internet [5].
- Integrated care of medical conditions (blocks 1, 2, 4, and 5). This category derives from Porter and Teisberg’s proposition of Integrated Practice Units, which are meant to create value around medical conditions, covering the complete cycle of care, including comorbidities [2]. In this category, standardized and non-standardized processes are mixed, but focused on a given medical condition or set of co-occurring conditions. However, risk stratification of patients typically shows that a big share of patients with a given condition are highly to moderately standardizable and their outcomes relatively predictable, while a minor share are more complex and non-standardizable. Chronic conditions, such as diabetes, heart failure, or chronic obstructive pulmonary disease, are more likely to benefit from this business model.
- Non-standardized processes of diagnosis and treatment (blocks 4 and 5). These are the typical processes that made the bulk of the workload at hospitals and doctors’ practices, when most health care was highly uncertain because of a lack of evidence. As scientific evidence allows for better predictability of outcomes in some areas of health care, standardization is more likely to be achieved. If standardized processes of care can also be separated from hospitals and doctors’ practices, these latter two business models will end up focusing on non-standardized, non-separable processes. It is obvious that these two pillars of health care delivery will not disappear, but it could be argued that their business models will be reshaped and their strategic focus will shift from “everything-to-everybody” to a narrower focus on those processes of care that exhibit a larger degree of uncertainty and a lower degree of separability.

In summary, uncertainty and information asymmetries give rise to a deeply-rooted supply-side mindset in health care delivery. The prevailing business models of hospitals and doctor’s practices emerged from this mindset and have not radically changed ever since. Standardizability and separability, along with patient-centeredness, allow for six new types of business models in addition to the traditional business models of hospitals and doctor’s practices. These radical changes in business models, more than just changing payment mechanisms, are more likely to yield real changes in how health care providers deliver more value for each dollar spent.

## Discussion

General hospitals usually cross-subsidize loss-making service lines with profit-making ones, so their sustainability depends critically on this compensatory scheme in order to distribute the heavy overhead burden [5]. This is one argument for hospitals to oppose the separation of service lines in freestanding facilities, particularly those that are more profitable.

However, it could be argued that, depending on how pricing is reshaped to reflect actual costs of production, this complex combination of cross subsidies can be reversed. Business models focused on highly standardized processes will be more able to bundle-price their services on a fee-for-outcome basis, and prices will be more likely to reflect marginal costs. General hospitals will be more able to price complex processes on a fee-for-service basis but also reflecting marginal costs. Therefore, competition will be more likely to result in higher value for money, just the way it happens in other markets.

Doctor’s practices, on the other hand, can also be reshaped into two different business models: one for highly standardized processes, such as minor ailments, routine follow-up of patients with chronic non-complex conditions, immunizations, and screening tests; and one for those patients that require further study or are more complex and non-standardized [5]. Standardized processes can be delegated to other health care workers without decreasing quality, and the most expensive and scarce workforce can be focused on those processes in which it is strictly necessary [16].

It can be expected that overall health system efficiency will benefit; hospitals and doctor’s practices will probably become more expensive, even though their prices reflect marginal costs. However, the other five business models will cause prices to decrease as a consequence of a more market-like competition. The net effect of both changes would be expected to be positive, i.e., net efficiency gains for the health system, or put another way, more value for money as a consequence of value-based competition. How does it impact equity and poverty? According to Scott et al. [17], universal coverage to improve access to care by the poor is not enough. They cite the experience of a conditional cash transfer program in India that improved access to maternity care and institutional delivery of babies, but did not improve maternal mortality rates. Therefore, the goal of universal coverage has to be rephrased to say “universal access to high-quality care for the poor.” Otherwise health policies aimed at universal coverage will be futile, health care will be less affordable and more inequitable, and health care spending will be more inefficient.

The quick answer to how business model innovations can improve equity in access to high-quality care is that a large part of the innovative business models that follow the lines of this article have emerged mostly in India and sub-Saharan Africa but also in other developing countries [18]. Surgery for cataracts and other eye conditions at Aravind Eye Care System and LV Prasad Eye Institute, birth attendance and maternity care at Life Spring, heart surgery and hip replacement at Narayana Hospitals, and cancer treatment at Health Care Global, are examples of low-cost high-quality services that are accessible to the poorest in India. Sala Uno is an application of the Aravind business model in Mexico. Penda Health and One Family Health are examples of primary care services in Africa. Medicall, Grand-Aides, and Aprofe are examples in Latin America and the United States [19]. It is clear that business model innovations in health care delivery hold a promise for affordable, high value-for-money solutions for the poorest.

## Summary

Innovations in health care delivery models are a necessary step to evolve towards a more market-like environment where competition among providers leads to more value for money. The traditional business models of general hospitals and doctor’s practices are not adequate for every kind of task, and this explains part of the sustainability problems of health care systems. New business models are emerging that shed light on this claim, but they show diverse degrees of innovation along the lines of standardizability and separability, as well as patient centeredness.
